# Transmission of Methicillin-Resistant *Staphylococcus aureus* CC398 from Livestock Veterinarians to Their Household Members

**DOI:** 10.1371/journal.pone.0100823

**Published:** 2014-07-25

**Authors:** Erwin Verkade, Marjolein Kluytmans-van den Bergh, Birgit van Benthem, Brigitte van Cleef, Miranda van Rijen, Thijs Bosch, Leo Schouls, Jan Kluytmans

**Affiliations:** 1 Laboratory for Microbiology and Infection Control, Amphia Hospital, Breda, The Netherlands; 2 Laboratory for Medical Microbiology and Immunology, St. Elisabeth Hospital, Tilburg, The Netherlands; 3 Amphia Academy Infectious Disease Foundation, Amphia Hospital, Breda, The Netherlands; 4 Centre for Infectious Disease Control Netherlands, National Institute for Public Health and the Environment, Bilthoven, The Netherlands; 5 Department of Medical Microbiology, VU University medical centre, Amsterdam, The Netherlands; University Medical Center Groningen, Netherlands

## Abstract

There are indications that livestock-associated MRSA CC398 has a reduced human-to-human transmissibility, limiting its impact on public health and justifying modified control measures. This study determined the transmissibility of MRSA CC398 from livestock veterinarians to their household members in the community as compared to MRSA non-CC398 strains. A one-year prospective cohort study was performed to determine the presence of MRSA CC398 in four-monthly nasal and oropharyngeal samples of livestock veterinarians (n  =  137) and their household members (n  =  389). In addition, a cross-sectional survey was performed to detect the presence of MRSA non-CC398 in hospital derived control patients (n  =  20) and their household members (n  =  41). *Staphylococcus aureus* isolates were genotyped by staphylococcal protein A (*spa*) typing and multiple-locus variable-number tandem repeat analysis (MLVA). Mean MRSA CC398 prevalence over the study period was 44% (range 41.6–46.0%) in veterinarians and 4.0% (range 2.8–4.7%) in their household members. The MRSA CC398 prevalence in household members of veterinarians was significantly lower than the MRSA non-CC398 prevalence in household members of control patients (PRR 6.0; 95% CI 2.4–15.5), indicating the reduced transmissibility of MRSA CC398. The impact of MRSA CC398 appears to be low at the moment. However, careful monitoring of the human-to-human transmissibility of MRSA CC398 remains important.

## Introduction

Infections with methicillin-resistant *Staphylococcus aureus* (MRSA) are associated with increased morbidity, mortality, and (healthcare) costs [1]–[2]. Traditionally, MRSA has been considered as a hospital-associated pathogen [3]–[4]. Since approximately 15–20 years, MRSA has expanded its territory to the community causing severe infections in previously healthy persons all over the world [5]. In 2003, a new clade of MRSA emerged in the Netherlands which was related to an extensive reservoir in pigs and cattle [6]–[7]. This so-called livestock-associated MRSA (LA-MRSA) mostly belongs to clonal complex 398 (MRSA CC398). National Dutch guidelines were adapted in June 2006, recommending persons in contact with live pigs or cattle to be screened upon hospital admission [8]. Since then the number of MRSA CC398 found in the Netherlands has dramatically increased. In 2010, 38% of all newly identified MRSA strains in humans in the Netherlands were of this type, up from 16% by the end of 2006 [9].

Several studies have reported the transmission of healthcare-associated MRSA (HA-MRSA) strains between patients and their household members, with transmission rates varying from below 10% up to 36% [10]–[14]. Recent surveys showed that MRSA CC398 was 4 to 6-fold less transmissible than other MRSA strains in a hospital-setting [15]–[17]. At present, the human-to-human transmissibility of MRSA CC398 in a community setting is still unclear. Considering the extensive reservoir in animals and people who work with livestock, the occurrence of MRSA CC398 in people who are not directly involved in farming is strikingly low. So far, there are no indications that MRSA CC398 has spread extensively into the general population. A cross-sectional survey in a livestock-dense region found that only 0.2% of adult individuals without livestock contact were positive for MRSA CC398 [18]. On the other hand, there are observations that proximity of farms is a potential risk factor, even in absence of direct contact between humans and animals [19]–[21]. In addition, a recent study suggest that incomplete cooking of contaminated meat can cause transmission [22].

Studying the human-to-human transmissibility of MRSA CC398 is hampered by the fact that the reservoir of MRSA CC398 is limited to the livestock agriculture setting, and that the majority of individuals working in this sector live on the farms together with their families, who mostly have direct animal contact themselves. Therefore, livestock veterinarians are an excellent group for studying human-to-human transmissibility of MRSA CC398 since their household members do not have direct contact with pigs or veal calves themselves.

The aim of this study was to determine the transmissibility of MRSA CC398 from livestock veterinarians to their household members compared to other MRSA strains in a community setting.

## Materials and Methods

### Study design and setting

A one-year prospective cohort study was conducted in Dutch livestock veterinarians and their household members. Individuals were sampled for the presence of MRSA and methicillin-susceptible *S. aureus* (MSSA) in the anterior nares and oropharynx every four months (total study period July 2008 through December 2009). In addition, to compare transmissibility of MRSA CC398 strains with MRSA non-CC398 isolates, a cross-sectional survey was performed in MRSA-positive hospital-based patients and their household members.

### Study population

Veterinarians associated with the Dutch Pig Health Department (VGV) were asked to participate in the study in April 2008. Control patients, defined as newly identified carriers of MRSA non-CC398, and their household members were recruited from a network of 16 hospitals and their affiliated microbiological laboratories between July 2009 and May 2011. Veterinarians and control patients were asked to take a nasal and oropharynx swab and to complete a questionnaire, which was used to determine the eligibility for the study. Subjects were eligible for participation if they (1) were aged between 18 and 65 years, (2) had one or more household members who were willing to participate, (3) did not live on a farm with pigs or veal calves, (4) did not have household members with professional pigs or veal calf contact, (5) were not treated for colonisation with MRSA in the previous three months, and (6) had provided written informed consent.

At baseline, veterinarians and control patients were visited at home and cultures were taken from the anterior nares and the oropharynx. Additional data were collected using a questionnaire that comprised information on age, gender, smoking, composition of the household, exposure to livestock, antibiotic treatment 4 months prior to sampling, and infections. Subsequently, veterinarians and their household members were asked to take nasal and oropharyngeal samples and complete a short questionnaire on the presence of active infections and antibiotic usage at 4, 8, and 12 months and return these by mail to the investigator. Appropriate transport material with Amies medium (Transwab, Medical Wire & Equipment), instructions for sampling and questionnaires were provided during the baseline home visit.

### Microbiological procedures

Nasal and oropharyngeal samples were separately plated on chromID *S. aureus* and chromID MRSA agar plates (bioMérieux, La Balme, France), and subsequently placed in two Mueller–Hinton (MH) broth supplemented with 6.5% NaCl. The overnight MH broth were separately subcultured onto both chromID *S. aureus* and chromID MRSA agar plates. All agar plates were read after 18–24 h incubation at 35–37°C according to manufacturer's instructions [23]. All cefoxitin resistant isolates were tested using a PCR for the presence of the *mec*A and *nuc* gene [24]–[25]. All *S. aureus* strains were genotyped by staphylococcal protein A (*spa*) typing [26] and multiple-locus variable-number tandem repeat analysis (MLVA) as described previously [27]. MLVA types (MTs) were clustered using a categorical clustering coefficient and a minimum spanning tree was constructed to display the relationships between the various MTs. MLVA complexes (MC) were assigned if two neighbouring MTs did not differ in more than one variable number tandem repeat (VNTR) locus and if at least five neighbouring MTs fulfilled this criterion.

### Definitions

Subjects were considered positive when either a nasal or an oropharynx swab harboured MRSA or MSSA. Subjects that were MRSA MC398 positive at all four sampling moments were defined as persistent MRSA MC398 carriers. Subjects that yielded MRSA MC398 in one to three samples out of all samples were defined as intermittent MRSA MC398 carriers and subjects that did not have any positive sample during the one-year study were defined as MRSA MC398 non-carriers.

A transmission event was confirmed when MRSA isolates of the same MLVA type were detected in veterinarians and in their household members on a specific sampling moment. A dyad was defined as a set of two household members, which could be MRSA positive or negative.

### Statistical analyses

All analyses were performed using SPSS 19.0 for Windows (SPSS Inc. Chicago, IL, USA). Differences in continuous variables between groups were tested with Student's t-test or Mann-Whitney U test when applicable and differences in categorical variables between groups were tested with the Pearson Chi-square test. Univariate backwards analysis for MRSA carriage were performed in a generalized estimated equations (GEE) model using a Poisson distribution with robust covariance estimators to calculate prevalence risk ratios (PRR) [28] with 95% confidence interval (95% CI).

### Ethics Statement

This study was approved by the medical ethics committee of the St. Elisabeth Hospital in Tilburg, the Netherlands (protocol number 0749). All participants had provided written informed consent and the reviewing medical ethics committee approved this consent procedure.

## Results

### Enrolment

#### Veterinarians

**Figure 1:**
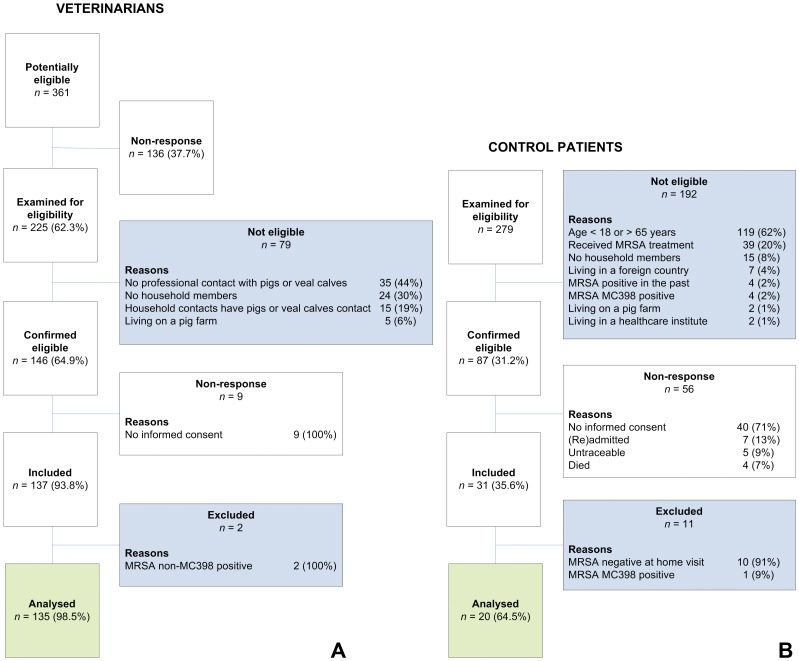
Flow chart of the recruitment of livestock veterinarians (A) and control patients (B).

Two hundred and twenty-five of 361 (62%) veterinarians responded and were examined for their eligibility. One-hundred forty-six veterinarians were eligible and after telephonic consultation, 137 veterinarians were included in the one-year follow-up study (Figure 1A). These veterinarians had a total of 389 household members (mean number of household members per veterinarian 2.8 persons).

#### Control patients

A total of 279 newly identified MRSA patients were reported to the central laboratory. After assessment of the inclusion – and exclusion criteria, 87 (31%) eligible patients were available (Figure 1B). After consultation by the investigator, 31 (36%) control patients were included and visited at home. Ten control patients that were visited at home were found to be MRSA-negative, and one control patient carried MRSA MC398. These eleven control patients were excluded from the analysis. A total of 20 (65%) control patients harboured MRSA non-MC398 and these subjects were included for the analysis. These patients had a total of 41 household members (mean number of household members per patient 2.1 persons).

### Results of MLVA and *spa*-typing

In total, 4246 samples were analysed in this study with the following distribution: 1086 samples from a total of 137 veterinarians and 3036 cultures originating from 389 household members. In addition, there were 42 samples from 21 control patients and 82 cultures originating from their household members. Throughout the study, only 5 out of 548 (response rate 99.1%) sampling moments from veterinarians and 38 out of 1556 (response rate 97.6%) of their household members were not received. Sensitivity analysis on the effect of missing samples had no relevant consequences on the conclusions (data not shown).

**Figure 2:**
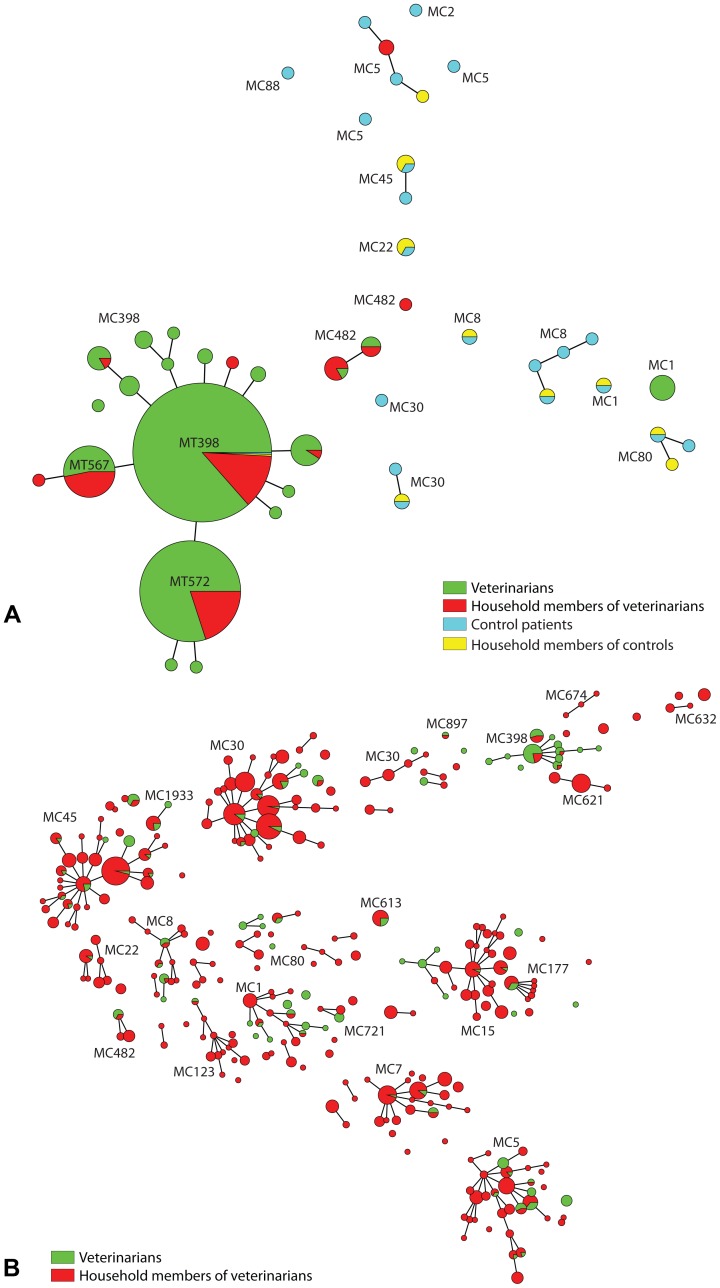
Genotypic relatedness of 482 MRSA (A) and 1308 MSSA (B) isolates derived from livestock veterinarians (green), household members of veterinarians (red), control patients (blue) and household members of control patients (yellow), represented as a minimum spanning tree based on MLVA types (MT), which are displayed as circles. The size of each circle indicates the number of isolates with this particular type. MLVA complexes are indicated in characters e.g. MC398 denotes MLVA complex 398.

A total of 1790 isolates were genotyped by *spa*-typing and MLVA: 365 MRSA and 211 MSSA strains from veterinarians, 84 MRSA and 1097 MSSA strains from household members of a veterinarian, 21 MRSA strains from control patients and 12 MRSA strains from household members of a control patient. In total, 341 different MTs belonging to 24 different MLVA complexes (MC) were found among the *S. aureus* isolates (Figure 2). Thirteen isolates (39%) from control patients and their household members had MCs that belonged to hospital-associated MRSA (HA-MRSA) strains. Two isolates (6%) were placed into MRSA MC398 and 18 isolates (45%) clustered into community-acquired MRSA (CA-MRSA). Eleven of these strains (33%) were Panton–Valentine leukocidin (PVL) positive. In contrast, none of the *S. aureus* MC398 strains originated from veterinarians and their household members were PVL-positive. Two-hundred twenty-two (12.4%) isolates did not belong to a known MLVA complex. The distribution of the different *spa*-types found in *S. aureus* MC398 isolates among veterinarians and their household members are summarized in Table 1. Two dominant *spa*-types, t011 and t108, accounted for 84% of all MRSA MC398 isolates and 4 *spa*-types were found only once. There were 54 MSSA isolates that belong to MC398; 42 isolates were derived from veterinarians (78%) and 12 isolates were recovered from household members (22%). Most of the MSSA strains had *spa*-type t034 (57%) or t081 (20%), which is in large contrast to the MRSA strains, where 9/430 (2.1%) MRSA MC398 isolates had *spa*-type t034 and none had *spa*-type t081.

**Table 1:** Distribution of *spa*-types of MRSA MC398 isolates (n = 430) and MSSA MC398 (n = 54) derived from livestock veterinarians and household members during the one-year study period.

	No. of MRSA MC398 isolates (%)			No. of MSSA MC398 isolates (%)			
Spa-type	Total	Veterinarians	Household members	Total	Veterinarians	Household members	P-value^a^
t011	241 (56.0%)	210	31	4 (7.4%)	3	1	<0.001
t108	121 (28.1%)	99	22	3 (5.6%)	3	none	<0.001
t567	17 (4.0%)	12	5	none	none	none	0.24
t1184	14 (3.3%)	3	11	none	none	none	0.38
t081	none	none	none	11 (20.4%)	6	5	<0.001
t034	9 (2.1%)	8	1	31 (57.4%)	25	6	<0.001
t1456	9 (2.1%)	8	1	none			0.61
t571	5 (1.2%)	5	none	2 (3.7%)	2	none	0.18
t1451	3 (0.7%)	2	1	none	none	none	1.00
t8333	3 (0.7%)	0	3	none	none	none	1.00
t899	2 (0.5%)	2	none	none	none	none	1.00
t3479	2 (0.5%)	2	none	none	none	none	1.00
t4652	none	none	none	2 (3.7%)	2	none	0.01
t1606, t2287, t4628, t6606	1 each (0.2%)	1 each	none	none	none	none	1.00
t5902	none	none	none	1 (1.9%)	1	0	0.11
Total	430 (100%)	355	75	54 (100%)	42	12	

a proportions of MRSA MC398 isolates vs proportions of MSSA MC398 isolates. A p-value ≤0.05 was considered statistically significant.

### Carriage of MRSA MC398 among veterinarians and their household members

Table 2 and 3 depict the distribution of all MRSA and MSSA isolates that belong to MLVA complex 398 or non-MC398 isolates found in nasal and oropharynx samples derived from veterinarians and their household members. From the 576 *S. aureus* isolates derived from veterinarians 365 isolates were MRSA (63%), while the 1181 *S. aureus* strains isolated from household members only 84 strains (7.1%) were methicillin-resistant. The vast majority of MRSA strains recovered from veterinarians (97%) and household members (89%) belonged to MC398. The mean prevalence of MRSA MC398 carriage among veterinarians was 44% (range 41.6–46.0%) and that of their household members was 4.0% (range 2.8–4.7%).

**Table 2:** Assignment to MLVA complex 398 (MC398), non-MC398 or no complex of MRSA and MSSA isolates derived from livestock veterinarians during the one-year study period.

Veterinarians (n = 137)	Mean prevalence % (range)	MC398 n (%)	non-MC398 n (%)	no complex n (%)	Total
MRSA isolates	44.6 (43.1–47.1)	355 (97.3) (97%)	10 (2.7)	0 (0)	365
MSSA isolates	26.3 (22.1–29.2)	42 (19.9)	125 (59.2)	44 (20.9)	211
All *S. aureus* isolates	70.9 (69.1–73.0)	397 (68.9) (68.(69%)	135 (23.4)	44 (7.6)	576

**Table 3:** Assignment to MLVA complex 398 (MC398), non-MC398 or no complex of MRSA and MSSA isolates derived from household members during the one-year study period.

Household members (n = 389)	Mean prevalence % (range)	MC398 n (%)	non-MC398 n (%)	no complex n (%)	Total
MRSA isolates	4.4 (3.1–5.0)	75 (89.3)	9 (10.7)	0 (0)	84
MSSA isolates	48.6 (45.9–50.3)	12 (1.1)	912 (83.1)	173 (15.8)	1097
All *S. aureus* isolates	53.0 (50.9–54.8)	87 (7.4)	921 (78.0)	173 (14.6)	1181

Table 4 shows the carrier state of veterinarians and their household members during the one-year study period. Two veterinarians had samples positive with MRSA non-MC398 strains and were excluded for further analysis. Forty veterinarians had four MRSA-positive test results (30%) and were defined as persistent MRSA MC398 carriers. Furthermore, 41 (30%) veterinarians were intermittent MRSA MC398 carriers and 54 (40%) veterinarians never carried MRSA. Altogether 36 from 386 non-exposed household members (9.3%), originating from 28 families (20.4%) carried MRSA MC398 intermittently. The prevalence of MRSA MC398 carriage in household members was statistically significantly higher for veterinarians with persistent MRSA MC398 carriage as compared to veterinarians with MRSA MC398 non-carriage (PRR 9.3; 95% CI 2.8–38.5) as well as veterinarians with intermittent MRSA MC398 carriage (PRR 2.1; 95% CI 1.0–4.6). MLVA genotyping data were not taken into account here. Results of confirmed transmission events are depicted in Table 4.

**Table 4:** Carriage of MRSA MC398 and transmission events among livestock veterinarians and their household members during the one-year study period.

	Total n (%)	Persistent MRSA MC398 carriage n (%)	Intermittent MRSA MC398 carriagen (%)	Non-carriersn (%)
Veterinarians	135 (100)	40 (29.6)	41 (30.4)	54 (40.0)
Household members	386	123	114	149
MRSA carriers among household members	36 (9.3)	23 (18.7)	10 (8.8)	3 (2.0)
Families with a transmission events	28 (20.7)	16 (40.0)	9 (22.0)	3 (5.6)
Confirmed transmission events to household members^a^	31 (8.0)	22 (17.9)	9 (7.9)	0 (0.0)

a A confirmed transmission events was defined as that veterinarian and household members were both MRSA-positive during one sampling moment with the same MLVA type.

### Transmissibility of MRSA MC398 compared to other MRSA strains

The prevalence of MRSA carriage among household members of livestock veterinarians and control patients was measured. The baseline characteristics of veterinarians and control patients are depicted in Table 5. There were significant differences between the two groups: gender, educational level, ethnicity, age, number of household members, and number of companion animals. The 59 veterinarians that were found MRSA MC398 positive at the initial sampling moment had a total of 180 household members. During the home visit 8 household members (4.4%) harboured MRSA MC398 with the same MLVA type as the index veterinarian, i.e. 4 partners and 4 children. This result was compared with the prevalence of MRSA non-MC398 in household members of 20 control patients, which had a total of 41 household members. Eleven household members (26.8%) carried MRSA non-MC398 with the same MTs as the control patients during the home visit. The MRSA MC398 prevalence in household members of veterinarians was significantly lower than the MRSA non-MC398 prevalence in household members of control patients (8/180 *vs.* 11/41, p<0.001). Hence, the transmission rate of MRSA non-MC398 strains is significantly higher than that of MRSA MC398 strains (PRR 6.0; 95% CI 2.4–15.5).

**Table 5:** Characteristics of livestock veterinarians with MRSA MC398 carriage and control patients with MRSA non-MC398 carriage at the initial sampling moment.

Characteristics	Veterinarians (n = 59)	Control patients (n = 20)	P-value
Age – median (IQR)	47.0 (41.0–52.0)	39.0 (24.3–57.8)	0.295
Male sex – no. (%)	55/59 (93.2)	7/20 (35.0)	<0.001
Smoking – no. (%)	10/58 (17.2)	4/15 (26.7)	0.467
Educational level^a^ – median (IQR)	6.9 (7.0–7.0)	3.6 (2.0–5.0)	<0.001
Born in the Netherlands – no. (%)	55/59 (93.2)	15/20 (75.0)	0.023
Age of household members – median (IQR)	16.0 (8.3–39.0)	34.0 (15.0–52.0)	<0.001
Number of household members – median (IQR)	3.0 (2.0–4.0)	1.5 (1.0–3.0)	0.006
Number of companion animals^b^ – median (IQR)	1.0 (0.8–2.0)	0.0 (0.0–1.0)	0.023

IQR: interquartile range (p25–p75); no.: number.

A P-value ≤0.05 was considered statistically significant.

a Highest educational level is a bachelor or master title and was valued with a maximum of 7.

b Total amount of cats and dogs in the household.

The MRSA MC398 prevalence per family was 13.6% for veterinarians and 45% for control patients (8/59 *vs.* 9/20, p = 0.009). At family level, the transmission rate of MRSA non-MC398 strains to other household members is significantly higher in comparison with MRSA MC398 (PRR 3.3; 95% CI 1.3–8.0). In addition, household member pairs (dyads) colonised with the same MLVA complex were identified to estimate the spread of different MRSA clades. In 59 families with MRSA MC398 colonisation, 8 MRSA MC398-positive member dyads out of 422 possible dyads (1.9%) were detected. By comparison, there were significant more concordant dyads among MRSA non-MC398 carriers (13/78 [16.7%], PRR 8.8; 95% CI 3.5–22.6).

## Discussion

Our prospective cohort study demonstrates that the mean prevalence of MRSA MC398 colonisation among household members of livestock veterinarians is relatively high (4.0%). None of these families were living on a farm or raising livestock. In total, 36 household members (9.3%), originating from 28 families (20.4%), harboured MRSA MC398 at least once during the one-year study period. These data confirm the results from a previous study performed in Germany in which an MRSA CC398 prevalence of 9.0% among household members of veterinarians was reported [29]. In our study, the prevalence of MRSA MC398 carriage among household members was shown to be highly dependent on the carrier state of the veterinarian. In addition, the prevalence of MRSA among household members was significantly higher for control patients carrying MRSA non-MC398 strains than for veterinarians carrying MRSA MC398 (PRR 6.0; 95% CI 2.4–15.5). These data suggest that MRSA MC398 spread less easily from humans with professional livestock contact to their household members than other MRSA non-MC398 isolates in a community setting. A possible explanation for this reduced transmissibility is that MRSA CC398 originates in humans as MSSA [30], and then spread to livestock, where it subsequently acquired the SCC*mec* cassette and methicillin-resistance. The jump of CC398 was also accompanied by the loss of phage-carried human virulence genes, making this clade less adapted to humans. In a hospital setting, Wassenberg and colleagues found that MRSA CC398 was 5.0 times less transmissible than other healthcare-associated MRSA (HA-MRSA) strains [15]. In general, transmission of MRSA within families seems to be common [10]–[14]. Several other studies showed high prevalences of MRSA among household members of individuals with HA-MRSA strains [11], [14], [31]–[34].

Guidelines underlying the Search & Destroy policy have been adapted in the Netherlands since 2006 based on conclusions from a case-control study [8]. Humans that work with live pigs and veal calves were defined as new risk populations for MRSA carriage and are now actively screened when admitted to a hospital. These guidelines were revised in December 2012, and all household members of confirmed MRSA patients have to be screened for MRSA on hospital admission. At present, household members of livestock veterinarians are not screened upon admission to a hospital. However, this study showed that they have a relatively high MRSA carriage in comparison to the Dutch general population [35]. Consequently, we advocate that household members of MRSA-positive veterinarians should also be screened for the presence of MRSA carriage upon hospital admission.

Despite the reduced human-to-human transmissibility of MRSA CC398, there are a few recent studies indicating that MRSA CC398 might have spread into the general population. A recent study found that MRSA with no link to established risk factors for acquisition, so-called MRSA of unknown origin (MUO), has now emerged [36]. Two distinct genotypic MUO groups were distinguished: MUO CC398 (26%) and MUO non-CC398 (74%), which suggests spread of MUO CC398, not by direct contact with livestock (pigs, veal calves), but through human-to-human transmission or by incomplete cooking of meat, but also by consumption of simultaneously prepared food products, such as salads, using contaminated kitchen equipment [22]. Furthermore, there are observations that proximity of farms is a potential risk factor, even in absence of direct contact between humans and animals [19]–[21].

To our knowledge, this study is the largest detailed survey among household members of livestock veterinarians. Unlike previous studies on transmissibility of MRSA CC398 [15], [29], [37], we performed this survey among veterinarians and their household members in a community setting for a prolonged period. Moreover, household members of veterinarians did not have livestock contact themselves during the study period. Therefore, we could estimate the human-to-human transmissibility of MRSA CC398 in detail. Thus the finding of 4.0% of household members carrying MRSA MC398 very likely represents the frequency of intrafamilial transmission. There are, of course, other possibilities of acquisition such as from pets and/or from horses. In addition, a cross-sectional survey to determine the transmissibility of MRSA CC398 in comparison with MRSA non-CC398 in the community was conducted. Here, the same inclusion - and exclusion criteria were used for control patients and veterinarians. Finally, we performed genotyping of all recovered *S. aureus* isolates, confirming the similarity between MRSA strains isolated from the veterinarians or control patients and their household members.

There are some limitations to our study. First, there was a large difference between response rate of veterinarians compared to control patients which may have caused selection bias. In addition, there were significant differences between gender, educational level and ethnicity, age and number of household members, and number of companion animals between control patients and veterinarians. However, to which extent these differences have influenced transmission rates remains unclear. Second, this study does not provide data on the exact transmission route to the household members (e.g. via direct physical contact or via contaminated household, i.e. doorknob, remote control, chairs, etc.). Third, MRSA CC398 isolates are hard to discriminate when using current molecular typing techniques, such as *spa*-typing and MLVA. This hampers studies that investigate possible transmission events and outbreaks caused by this MRSA clade. Finally, a minor limitation is that the results are somewhat outdated because data collection started in August 2008. However, we believe that human-to-human transmissibility of MRSA CC398 has not changed much in 5 years. Since this was a prospective cohort study with one-year follow-up it was inevitable to report the results after several years.

In summary, MRSA CC398 colonisation was common among household members of livestock veterinarians and this was shown to be highly dependent on the carrier state of the veterinarian. Moreover, the transmissibility of MRSA CC398 in the community setting was found to be substantially lower than that of MRSA non-CC398 strains. Therefore, we believe that screening of household members of MRSA-positive veterinarians upon hospital admission is justified and that the current Dutch MRSA guidelines can be maintained. The present situation is a widespread resistant bacterium with an enormous reservoir in livestock. The impact of MRSA CC398 appears to be low at the moment. However, when MRSA CC398 acquires genetic elements harbouring virulence factors it may pose a significant public health problem in the future. Careful monitoring of the human-to-human transmissibility of MRSA CC398 is therefore important.
